# Integrated Multi-Omics Analysis of the Developmental Stages of *Antheraea pernyi* Pupae: Dynamic Changes in Metabolite Profiles and Gene Expression

**DOI:** 10.3390/insects16070745

**Published:** 2025-07-21

**Authors:** Shuhui Ma, Yongxin Sun, Yajie Li, Xuejun Li, Zhixin Wen, Rui Mi, Nan Meng, Xingfan Du

**Affiliations:** Liaoning Ocean and Fisheries Science Research Institute, Liaoning Academy of Agricultural Sciences, Dalian 116023, China; msh200461@163.com (S.M.); sunyongxin1977@163.com (Y.S.); lyj81103392@163.com (Y.L.); dllixuejun@126.com (X.L.); hansonwen2003@163.com (Z.W.); lianyi7432@sina.com (R.M.); mona0408@163.com (N.M.)

**Keywords:** *Antheraea pernyi* pupae, non-targeted metabolomics, transcriptomics, developmental stages

## Abstract

This study integrated non-targeted metabolomics and transcriptomics to investigate dynamic changes in *Antheraea pernyi* pupae across five developmental stages. Metabolomic analysis identified 1246 metabolites, primarily organic acids, lipids, heterocyclic compounds, and oxygen-containing organics. Principal component analysis revealed stage-specific metabolic profiles: amino acid derivatives (pyruvate, proline, lysine) declined, while pyrimidines (cytidine, uridine, β-alanine) and monosaccharides (glucose, mannose) increased. 18β-glycyrrhetinic and ursolic acids accumulated significantly in the middle and late stages. Transcriptomic analysis identified 7230 differentially expressed genes (DEGs), with 366, 1705, and 5159 significantly differentially expressed genes in the T1, T3, and T5 comparison groups, respectively. KEGG enrichment highlighted ABC transporters, amino acid/pyrimidine metabolism, and tyrosine pathways as developmentally critical, with aminoacyl-tRNA biosynthesis upregulated in later phases. Integrated multi-omics analysis revealed coordinated shifts in metabolites and genes across developmental phases, reflecting dynamic nutrient remodeling during pupal maturation. This study systematically delineates the molecular transitions driving pupal development in *Antheraea pernyi* pupae, uncovering conserved pathway interactions and mechanistic insights into nutrient metabolism. These findings provide a scientific foundation for leveraging pupal resources in functional food innovation and bioactive compound discovery for pharmaceutical applications.

## Simple Summary

We tracked metabolic and genetic changes across five developmental stages of *Antheraea pernyi* (oak silkworm) pupae using combined metabolomics and transcriptomics. Key findings show that critical bioactive substances such as 18β-glycyrrhetinic acid and ursolic acid (anti-inflammatory triterpenoids) surged 1677-fold and 13,126-fold in the late stages, which is associated with ABC transporters. Metabolic patterns also shifted from early-stage amino acid catabolism to late-stage pyrimidine/nucleotide synthesis, driven by coordinated gene–metabolite networks like aminoacyl-tRNA biosynthesis. Additionally, glutathione accumulation peaked at 1976-fold in the late phases, acting as a key redox buffer during metamorphosis. This study decodes the molecular basis of nutrient dynamics in silkworm pupae, supporting their application as sustainable sources for functional foods and pharmaceuticals.

## 1. Introduction

The oak silkworm (*Antheraea pernyi*), a member of the order Lepidoptera, family Saturniidae, and genus Antheraea, undergoes complete metamorphosis with a life cycle encompassing egg, larva, pupa, and adult stages. Notably, its pupal phase serves dual roles as the overwintering form and as a nutrient-dense reservoir rich in proteins, lipids, vitamins, and bioactive compounds, endowing it with exceptional nutritional and medicinal value. This species holds significant economic importance in China, where it is cultivated as a vital insect resource [1]. Annual production of oak silkworm pupae in China reaches tens of thousands of tons, widely utilized in food processing, health products, and pharmaceutical raw materials, underscoring its substantial economic importance [2]. Recent studies have identified functional components in oak silkworm pupae, such as the triterpenoids 18β-glycyrrhetinic acid and ursolic acid, which exhibit anti-inflammatory, immunomodulatory, and antioxidant properties [3]. Additionally, pupae contain essential amino acids, nucleotide derivatives, and polysaccharides that regulate human metabolism and enhance immunity [4]. Nutrient accumulation in pupae exhibits significant temperature dependence. Research has demonstrated that metabolite composition and bioactive content undergo dynamic changes under varying accumulated temperature units (ATUs), providing critical insights for targeted resource utilization [5]. Diapause-maintenance phase pupae were selected as controls (CK) because they represent a metabolically suspended state [6], providing a null reference for developmental transitions.

Advancements in systems biology have made multi-omics technologies such as metabolomics, transcriptomics, and proteomics pivotal tools for deciphering dynamic nutrient profiles and molecular mechanisms in food science [7,8,9]. By integrating multi-layered data, these approaches comprehensively reveal synergistic regulatory networks linking gene expression, protein function, and metabolic pathways [10,11], offering theoretical foundations for functional food development and bioactive compound discovery. For instance, integrated multi-omics analyses have been extensively applied in the domesticated silkworm (*Bombyx mori*) to elucidate physiological, biochemical, and developmental mechanisms, reinforcing its role as a model organism [12,13]. Similarly, in *Manduca sexta*, multi-omics analyses have revealed hormonal regulation of pupal diapause and immune activation [14]. These works demonstrate the power of integrated omics in uncovering conserved pathways governing insect development. In contrast, studies on *A. pernyi* remain fragmented, limited to single-omics approaches: proteomics has differentiated diapause and non-diapause pupae [15], and transcriptomics has dissected viral infection mechanisms [16]. Systematic multi-omics investigations into nutrient dynamics during *A. pernyi* pupae development is absent, leaving metabolic pathway regulation and bioactive accumulation poorly understood.

This study pioneers an integrated metabolomics and transcriptomics strategy to systematically unravel nutrient dynamics across five developmental stages of *A. pernyi* pupae. By synthesizing multi-omics data, we delineate metabolite accumulation patterns, key gene expression profiles, and protein regulatory networks [17], elucidating the molecular basis of nutrient remodeling. Our findings not only provide a theoretical framework for the high-value utilization of *A. pernyi* pupae, including functional food innovation and natural drug discovery, but also promote the research of insect developmental biology and metabolic regulation [18,19].

## 2. Materials and Methods

### 2.1. Materials

The *A. pernyi* pupae used in this study were of the bivoltine variety 9906, obtained from the Sericulture Research Institute of Fengcheng, Liaoning Province, China. The pupae were reared under controlled environmental conditions (25 °C, 70% relative humidity, and a 12 h light/dark cycle). Samples were collected at six developmental stages: The CK group consisted of diapause-maintenance phase pupae, identified by stable weight (<2% change over 14 days), absence of cuticle pigmentation, and metabolic quiescence (O_2_ consumption rate < 0.05 μL/h/mg) [6]. Other groups were T1 (early stage), T2 (mid-early stage), T3 (mid-early), T4 (mid-late stage), and T5 (late stage). Each stage was defined based on accumulated temperature units (ATUs), with T1 at 0 °C, T2 at 30 °C, T3 at 60 °C, T4 at 120 °C, and T5 at 150 °C. The samples were immediately frozen in liquid nitrogen and stored at −80 °C until further analysis.

### 2.2. Metabolomics Analysis

#### 2.2.1. Metabolite Extraction

Samples were pulverized in liquid nitrogen, and 100 mg portions were weighed. Each sample was supplemented with 200 µL pre-cooled water and 800 µL ice-cold methanol/acetonitrile mixture (1:1, *v*/*v*). Following homogenization, the mixture was sonicated in an ice bath for 60 min and then incubated at −20 °C for 1 h to facilitate protein precipitation. The samples were centrifuged at 16,000× *g* for 20 min at 4 °C, and the resulting supernatant was collected and dried using a high-speed vacuum concentrator. For mass spectrometric analysis, the dried residues were re-dissolved in 100 µL acetonitrile–water solution (1:1, *v*/*v*) and centrifuged at 14,000× *g* for 15 min at 4 °C, and the supernatant was taken for injection.

#### 2.2.2. Chromatographic Separation

Samples were stored in an autosampler at 4 °C throughout the analysis. Chromatographic separation was carried out on an Agilent 1290 Infinity LC UHPLC system fitted with a HILIC column (Santa Clara, CA, USA). The injection volume was 5 µL, column temperature was maintained at 25 °C, and the flow rate was set to 0.3 mL/min. Mobile phase A was composed of water containing 25 mM ammonium acetate and 25 mM ammonia, while mobile phase B was acetonitrile. The gradient elution program was as follows: 95% B was maintained from 0 to 0.5 min; B was linearly decreased from 95% to 65% between 0.5 and 7 min; further linearly reduced from 65% to 40% over 7–9 min; kept at 40% from 9 to 10 min; linearly increased from 40% to 95% during 10–11.1 min; and held at 95% from 11.1 to 16 min. Quality control (QC) samples were incorporated into the sample sequence to oversee system stability and validate the reliability of experimental data.

#### 2.2.3. Mass Spectrometry Acquisition

Post UPLC separation, each sample was analyzed in both positive and negative ion modes via electrospray ionization (ESI) using a Triple-TOF 5600 mass spectrometer (AB SCIEX, Framingham, MA, USA). ESI source parameters were configured as follows: Ion Source Gas 1 (Gas1) and Gas 2 (Gas2) at 60 psi each; Curtain gas (CUR) at 30 psi; source temperature at 600 °C; Ion Spray Voltage Floating (ISVF) at ±5500 V (for both modes). The TOF MS scan covered an *m*/*z* range of 60–1200 Da with an accumulation time of 0.15 s/spectrum, and product ion scans ranged from 25 to 1200 Da with an accumulation time of 0.03 s/spectrum. High-sensitivity information-dependent acquisition (IDA) mode was employed for secondary mass spectrometry, with the following settings: declustering potential (DP) at ±60 V (both modes); collision energy at 30 eV; IDA parameters set to exclusion of isotopes within 4 Da and monitoring of 6 candidate ions per cycle.

#### 2.2.4. Data Preprocessing

Raw data were transformed and processed using the XCMS tool within MSDIAL software (1.0.0.0.) for peak alignment, retention time calibration, and peak area extraction. Metabolite identification was accomplished by matching exact mass (<25 ppm) and MS/MS spectra against public repositories (HMDB, MassBank) and an in-house standard library (Shanghai Bioprofile Technology Co., Ltd., Shanghai, China). Data points with over 50% missing values in any group were eliminated. Integrated positive and negative ion peaks were subjected to pattern recognition analysis using SIMCA-P 14.1 software (Umetrics, Umeå, Sweden). After unit variance scaling (UV) preprocessing, multivariate statistical analyses were conducted, including unsupervised principal component analysis (PCA), supervised partial least squares discriminant analysis (PLS-DA), and orthogonal partial least squares discriminant analysis (OPLS-DA). Differential metabolites were filtered based on *VIP* values (from multivariate statistics), *p*-values (from univariate statistics), and fold changes. Metabolites with *VIP* > 1 and *p* < 0.05 were deemed significant and subjected to pathway analysis using the KEGG database.

### 2.3. Transcriptomics Analysis

#### 2.3.1. RNA Extraction

Total RNA was isolated and purified from samples using Trizol reagent (Invitrogen, Carlsbad, CA, USA) following the manufacturer’s instructions. RNA concentration and purity were measured using a NanoDrop 2000 (NanoDrop, Wilmington, DE, USA), and integrity was assessed with an Agilent 2100 Bioanalyzer (Santa Clara, CA, USA), with only samples having RIN values > 7.0 being utilized.

#### 2.3.2. Construction of RNA-Seq Libraries

mRNA was enriched from total RNA using oligo(dT) magnetic beads, then fragmented into small segments with divalent cations at 85 °C. The fragmented RNA was reverse-transcribed into cDNA, followed by synthesis of U-labeled second-stranded DNAs using *E. coli* DNA polymerase I, RNase H, and dUTP. A-base overhangs were added to the blunt ends of each strand to enable ligation with indexed adapters (containing T-base overhangs). After ligation of single- or dual-index adapters, size selection was performed using AMPure XP beads (Brea, CA, USA). Following treatment of U-labeled second-stranded DNAs with heat-labile UDG enzyme, the ligated products were amplified via PCR under the following conditions: initial denaturation at 98 °C for 30 s; 14 cycles of denaturation at 98 °C for 15 s, annealing at 60 °C for 30 s, and extension at 72 °C for 30 s; final extension at 72 °C for 5 min. The average insert size of the final cDNA library was 350 bp (±50 bp). Paired-end sequencing was executed on an Illumina HiSeq X-Ten platform (LC Bio, Hangzhou, China) according to the vendor’s protocol.

#### 2.3.3. Bioinformatics Analysis

Raw reads were processed with Cutadapt to remove adapter contaminants, low-quality bases, and ambiguous bases [20]. Sequence quality was verified using FastQC (http://www.bioinformatics.babraham.ac.uk/projects/fastqc/, accessed on 16 June 2021). Reads were aligned to the mouse genome GRCm38 (Ensemble92) using Hisat2 [21], and mapped reads for each sample were assembled with StringTie [22]. Transcriptomes from all samples were merged to reconstruct a consensus transcriptome.

## 3. Results

### 3.1. Metabolomics Analysis

#### 3.1.1. Overall Metabolomics Analysis of Samples at Different Growth Stages

Based on the LC-MS/MS detection platform and the self-built database of Shanghai Bioprofile Technology Co., Ltd. (Shanghai, China) as well as public database information, a total of 1246 metabolites were detected in samples from different growth stages. Excluding the 694 metabolites that were not classified, 552 metabolites were categorized into 13 classes according to their characteristics (Table 1). Organic acids and their derivatives were the most abundant, with 135 metabolites accounting for 24.46% of the total. The next most abundant classes were organic heterocyclic compounds and lipids/lipid-like molecules, representing 18.48% and 13.22% of the total, respectively. Throughout the developmental stages from T1 to T5, the metabolites that were upregulated included 4-methylpyrimidine, purine, β-alanine, lactate, mannitol, potassium sorbate, dihydroartemisinin, berberine, flonicamid, formoterol, indigo, mulberroside, penicillin G, methyl quercetin, tetracycline antibiotics, etc. The metabolites that were downregulated included 1,1,2-trimethylbenzene-1H-benz[e]indole, methyl anthrone, piperidinone, acrylic trimellitate, ascorbic acid, nervonic acid, oleoyl sarcosine, phosphatidylserine 16:0–20:4, glycerophosphocholine, isopropanol, sodium dodecyl sulfate, diphenyl phosphate, citric acid, disodium D-glucose-1-phosphate, ergosterol, Furosemide, pyridoxal phosphate, syringin-3-O-galactoside, sodium tetradecyl sulfate, etc.

#### 3.1.2. Principal Component Analysis

**Figure 1:**
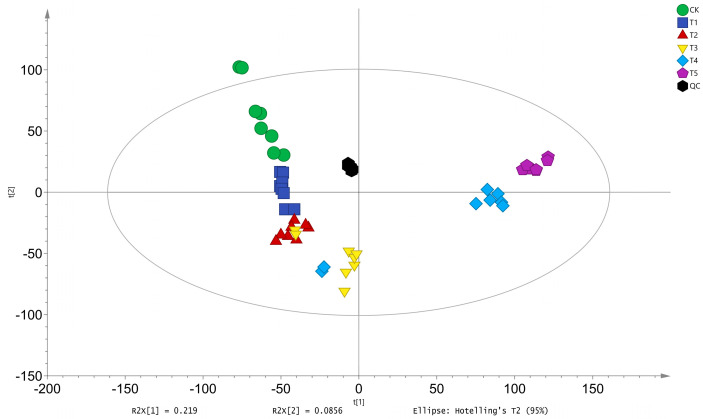
Principal component analysis of metabolome samples at different developmental stages of *A. pernyi* pupae. Note: CK—diapause period of *A. pernyi* pupae (deep diapause state with arrested development and minimal metabolic activity); T1—early development stage of *A. pernyi* pupae; T2—mid-early development stage of *A. pernyi* pupae; T3—mid-stage of *A. pernyi* pupae; T4—mid-late stage of *A. pernyi* pupae; T5—late stage of *A. pernyi* pupae; QC—All samples mixed equally. “R^2^×[1] and R^2^×[2] denote the proportion of variance explained by the 1st and 2nd principal components (PC1 and PC2) in the PCA.

Principal component analysis (PCA) is an unsupervised data analysis method that recombines all identified metabolites into a set of new composite variables. It selects a few composite variables from the analysis to reflect as much information as possible from the original variables, thereby achieving dimensionality reduction. Additionally, PCA of metabolites can reflect the overall variability between and within sample groups. Analysis of the PCA score plots of *A. pernyi* pupae at different developmental stages revealed that the points within each group were clustered closely, indicating a reliable model. The PCA points for the CK and T1 groups were distributed in the second quadrant, while the T2 and T3 groups were in the third quadrant, the T4 group in the fourth quadrant, and the T5 group in the first quadrant. This distribution indicates significant differences in metabolites among *A. pernyi* pupae at different developmental stages (Figure 1).

#### 3.1.3. Univariate Statistical Analysis of Differential Metabolites at Different Developmental Stages

**Figure 2:**
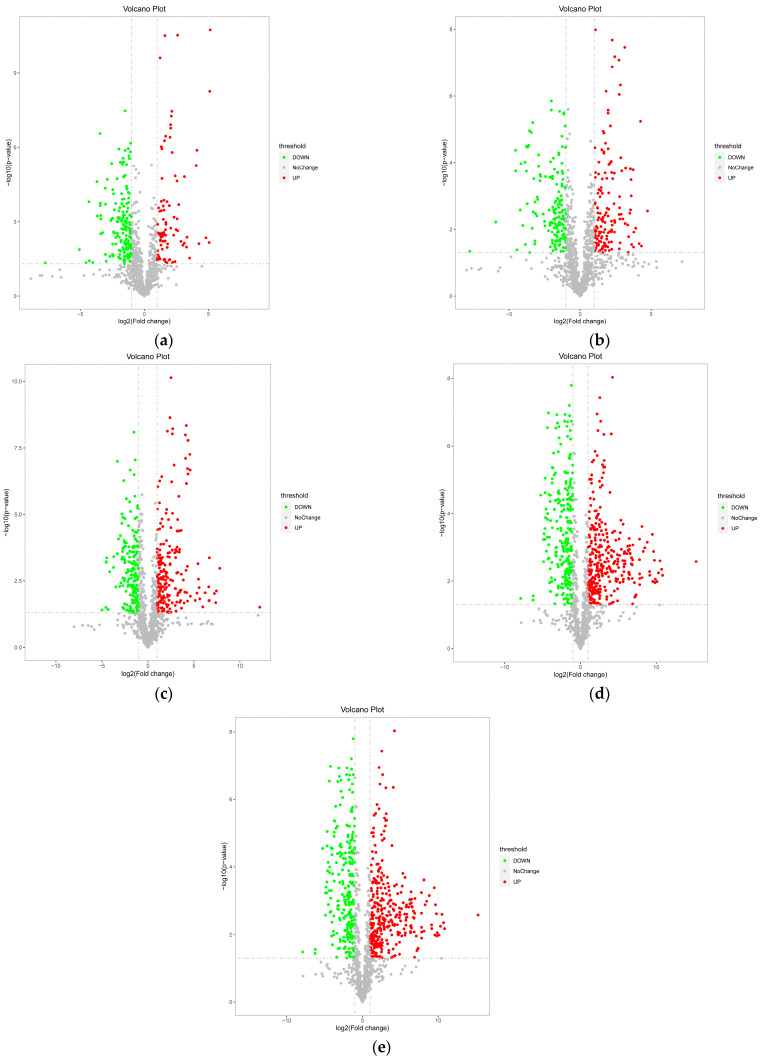
Volcano plots of differential metabolites in *A. pernyi* pupae at different developmental stages ((**a**): T1 vs. CK; (**b**): T2 vs. CK; (**c**): T3 vs. CK; (**d**): T4 vs. CK; (**e**): T5 vs. CK). Note: The two vertical dotted lines represent the log_2_ (fold change) thresholds (e.g., set as ±1 in this analysis). Genes with log_2_ (fold change) > 1 (right of the right—hand dotted line) are labeled as up—regulated (red dots), and those with log_2_ (fold change) < −1 (left of the left—hand dotted line) are down—regulated (green dots). Dots between the lines show no significant fold change.

Univariate analysis can intuitively display the significance of metabolite changes between two sample groups. In this study, a screening criterion of *FC* (fold change) > 2 or *FC* < 0.5 and *p*-value < 0.05 was used for univariate analysis to intuitively show the significance of metabolite changes between two sample groups. Figure 2 shows the volcano plots of T1–T5 vs. CK as examples, where red points represent upregulated metabolites and green points represent downregulated metabolites. Table 2 shows the comparison of the number of significant differential metabolites during the development of *A. pernyi* pupae. It can be seen that with the increase in accumulated temperature and development of the pupae, the number of differential metabolites increases significantly, and the changes in metabolite accumulation are more pronounced in the later stages of development. The number of downregulated products remains relatively stable across different stages, while the number of upregulated products increases with the increase in accumulated temperature.

#### 3.1.4. Hierarchical Clustering Analysis

**Figure 3:**
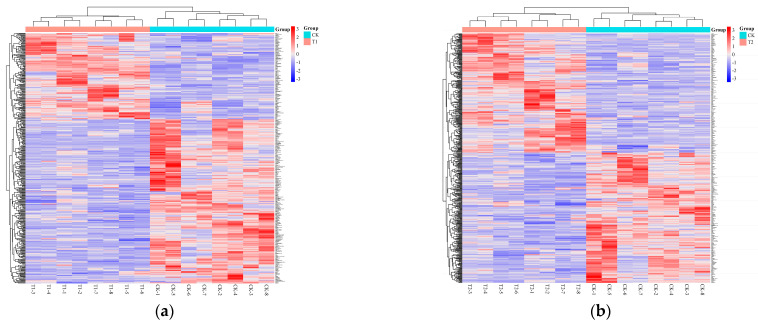

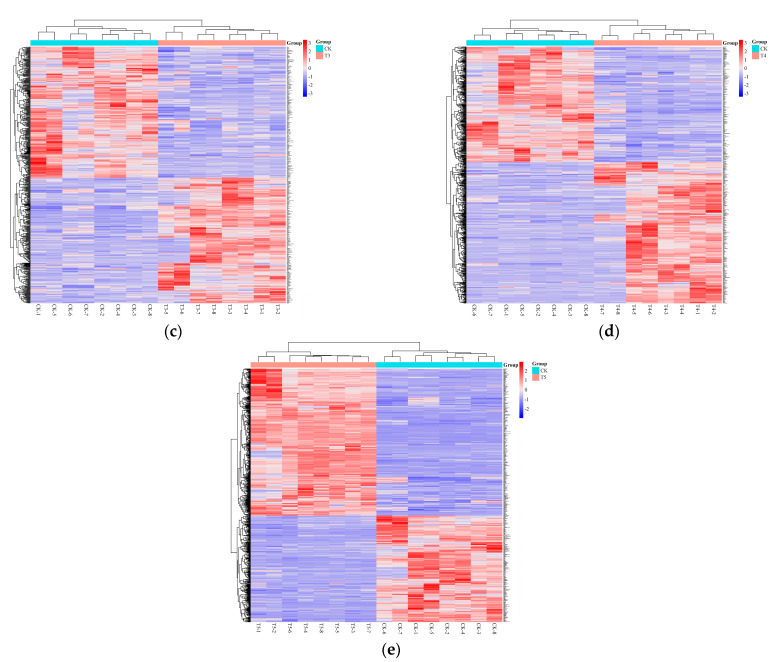
Hierarchical clustering analysis of differential metabolites at different developmental stages of *A. pernyi* pupa ((**a**): T1 vs. CK; (**b**): T2 vs. CK; (**c**): T3 vs. CK; (**d**): T4 vs. CK; (**e**): T5 vs. CK). The original figures are available in the Supplementary Materials.

To more intuitively display the differences in metabolite content among different samples and the relationships between samples, hierarchical clustering was performed based on the content of significantly different metabolites. Generally, when the selected metabolites are reasonable and accurate, samples from the same group can cluster together in the same cluster. Moreover, metabolites clustered together tend to have similar expression patterns, suggesting that they may be involved in closely related steps in metabolic processes. Figure 3 shows the hierarchical clustering results of significant differential metabolites for the comparison groups T1–T5 and the CK group, with colors corresponding to different expression levels: red for upregulated and blue for downregulated metabolites. As shown in the figure, metabolites exhibit high expression at each developmental stage of the *A. pernyi* pupae. There are no significant differences in metabolites between pupae with one stage of accumulated temperature difference. However, significant differences in metabolites are observed between the early, middle, and late stages of the pupae.

#### 3.1.5. Significant Differential Metabolites at Different Developmental Stages

In general metabolomics analysis, the Variable Importance in Projection (VIP) values obtained from the OPLS-DA model are used to measure the impact and explanatory power of each metabolite’s expression pattern on the classification and discrimination of samples, thereby identifying biologically significant differential metabolites. In this study, metabolites with VIP > 1 and a *p*-value (using One-Way ANOVA for multiple comparisons) less than 0.05 were considered significantly different. The analysis results showed that glycyrrhetinic acid and ursolic acid had fold change (FC) values of 1677.51 and 13,126.44, respectively, in the T4 vs. CK and T5 vs. CK stages, indicating significant increases in content during the middle and late stages.

#### 3.1.6. KEGG Metabolic Pathway Analysis

**Figure 4:**
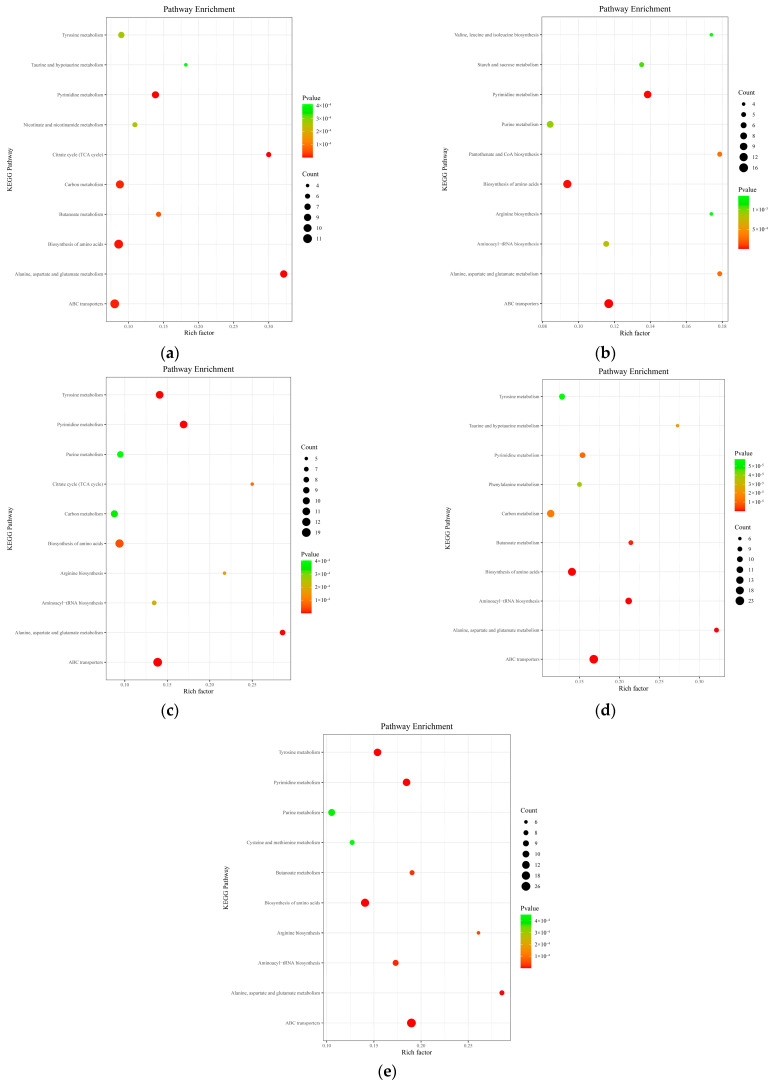
Bubble diagram of KEGG metabolism pathway of *A. pernyi* pupae’s differential metabolites (Top 10) ((**a**): T1 vs. CK; (**b**): T2 vs. CK; (**c**): T3 vs. CK; (**d**): T4 vs. CK; (**e**): T5 vs. CK). Note: Abscissa represents the degree of enrichment; ordinate represents the pathway name of the top 10; color of the point represents the *p*-value; size of the point represents the number of different metabolites enriched.

As illustrated in Figure 4, significant differential metabolites from *A. pernyi* pupae at different developmental stages were subjected to KEGG pathway enrichment analysis. Throughout the developmental stages, differential metabolites were significantly enriched in four metabolic pathways: ABC transporters, biosynthesis of amino acids, pyrimidine metabolism, and tyrosine metabolism. In addition to these, during the middle and late stages (T3–T5 groups), metabolites were also significantly enriched in the aminoacyl-tRNA biosynthesis pathway. At different stages, other involved pathways included purine metabolism; butanoate metabolism; taurine and hypotaurine metabolism; arginine biosynthesis; carbon metabolism; alanine, aspartate, and glutamate metabolism; nicotinate and nicotinamide metabolism; and the citric acid cycle (TCA cycle).

Compared with the CK group, as the effective accumulated temperature increased in the early developmental stage (T1 group), there were five amino acid metabolites that were upregulated and six that were downregulated. In the middle developmental stage (T3 group), 2 metabolites were upregulated and 10 were downregulated. In the late developmental stage (T5 group), 7 metabolites were upregulated and 11 were downregulated. Throughout the developmental stages of *A. pernyi* pupae, more amino acid metabolites were downregulated than upregulated. Compared with the CK group, in the T1 group, the significantly increased metabolites were tyrosine, methionine, alanine, and sedoheptulose-7-phosphate, while the significantly decreased metabolites were pyruvate, dimethyl-2-oxoglutarate, cysteine, and glutamine. In the T3 group, the significantly increased metabolites were tyrosine and citrulline, while the significantly decreased metabolites were pyruvate, dimethyl-2-oxoglutarate, proline, lysine, valine, and cysteine. In the T5 group, the significantly increased metabolites were tyrosine, methionine, citrulline, and glutamate, while the significantly decreased metabolites were pyruvate, dimethyl-2-oxoglutarate, proline, glycine, and lysine. The amino acid metabolic pathway exhibited an overall suppression. It can be observed that tyrosine was consistently upregulated throughout the developmental process.

In pyrimidine metabolism, compared with the CK group, the T1 group had three upregulated metabolites (β-alanine, deoxycytidine, and 5-methylcytosine) and seven downregulated metabolites (L-glutamine, uridine, uridine monophosphate, cytosine, cytidine monophosphate, and urea). The T3 group had six upregulated metabolites (β-alanine, uridine, uracil, cytosine, 5-methylcytosine, and orotate) and six downregulated metabolites (L-glutamine, cytidine, uridine monophosphate, urea, and 3-hydroxypropionic acid). The T5 group had 10 upregulated metabolites (β-alanine, uridine, pseudo-uridine, orotate, cytidine monophosphate, cytidine, deoxycytidine, and urea) and two downregulated metabolites (cytosine and 3-hydroxypropionic acid). Pyrimidine metabolism demonstrated enhanced developmental stage-dependent regulation, with the number of upregulated metabolites increasing to 10 in the T5 stage and key metabolites like β-alanine and uridine persisting across multiple developmental phases, potentially serving as precursors for nucleic acid synthesis during pupal development.

In the ABC transporter pathways, compared with the CK group, the T1 group had six upregulated metabolites (glucose, α-glucoside, mannitol, rhamnose, mannose, and histidine) and six downregulated metabolites (glutamine, aspartate, arginine, urea, N-acetylglucosamine, and nucleoside). The T3 group had six upregulated metabolites (mannitol, rhamnose, hydroxyproline, α-glucoside, glutathione, and xylitol) and 15 downregulated metabolites (histidine, lysine, arginine, glutamine, urea, N-acetylglucosamine, nucleoside, etc.). The T5 group had 13 upregulated metabolites (glucose, α-glucoside, mannose, fructose, N-acetylglucosamine, phosphate, hydroxyproline, proline, glutathione, nucleoside, etc.) and 16 downregulated metabolites (glutamine, histidine, lysine, arginine, glycine, ornithine, biotin, etc.). The accumulation of sugars, amino acid transport-related substances (e.g., fructose, proline), and glutathione may provide antioxidant protection and material reserves for the late pupal developmental stage.

### 3.2. Transcriptomics Analysis

#### 3.2.1. Analysis of Differentially Expressed Genes in Different Developmental Stages of *A. pernyi* Pupae

During the developmental process of *A. pernyi* pupae, the number and direction of differentially expressed genes (DEGs) across stages compared with the control (CK) exhibited significant dynamic changes. In the early stage (T1), 366 DEGs were identified, with 74.9% (274 genes) being downregulated. These downregulated genes were predominantly associated with amino acid metabolism and basal energy catabolism. As development progressed, the total number of DEGs increased substantially to 1705–5159 in the middle to late stages (T3–T5), accompanied by a gradual rise in the proportion of upregulated genes (e.g., 2693 upregulated genes, accounting for 52.2% in T5). These upregulated genes were significantly enriched in pathways related to carbohydrate metabolism, ribosome biogenesis, and immune defense. For instance, in the T5 stage, upregulated genes were primarily clustered in redox enzyme activity and purine nucleotide metabolism, while downregulated genes were linked to amino acid degradation, reflecting a shift toward enhanced energy metabolism and prioritized structural protein synthesis during later developmental phases. Overall, the transcriptional dynamics indicated a transition from catabolism-dominated processes in the early stages to anabolism and stress adaptation in middle to late stages, concomitant with a marked increase in the complexity of the gene expression patterns.

#### 3.2.2. GO Functional Enrichment Analysis

**Figure 5:**
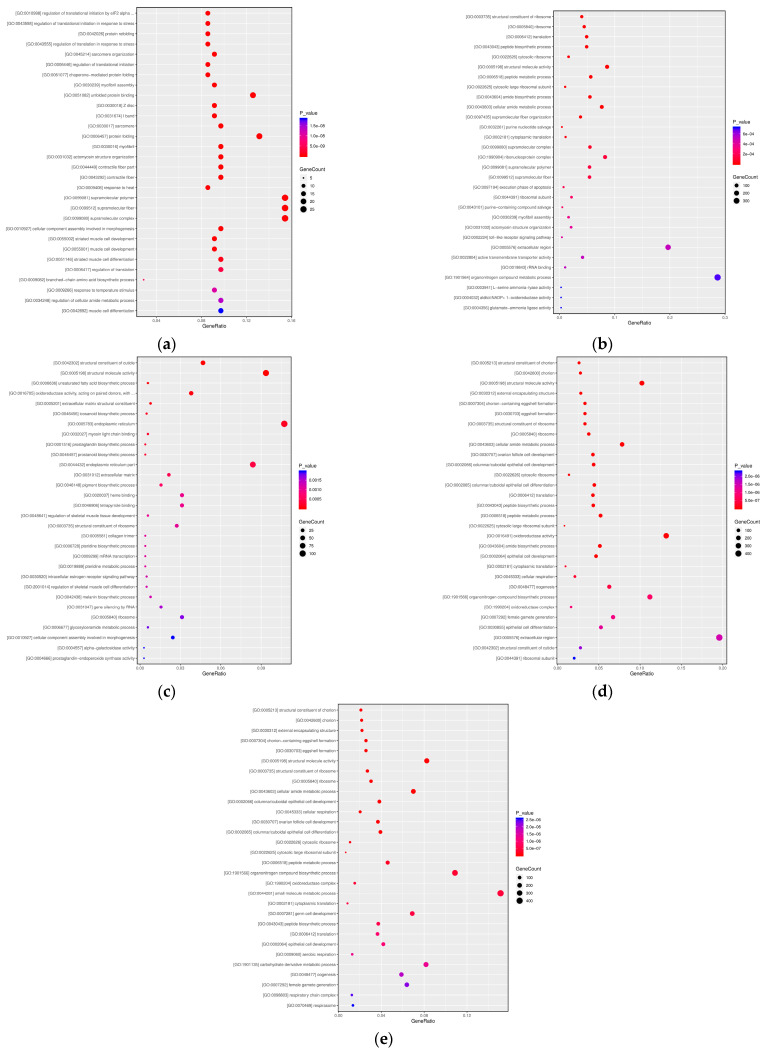
Transcriptome GO analysis of *A. pernyi* pupae at different developmental stages ((**a**): T1 vs. CK; (**b**): T2 vs. CK; (**c**): T3 vs. CK; (**d**): T4 vs. CK; (**e**): T5 vs. CK).

Transcriptomic analysis of *A. pernyi* pupae across the T1–T5 developmental stages revealed dynamic molecular regulatory features (Figure 5). During T1, stress response dominated, with significant enrichment of eIF2α phosphorylation-mediated translational regulation (GO:0010998, *P adjust* < 0.01) and myofibril organization (GO:0045214, *P adjust* < 0.01), alongside coordinated activation of heat shock proteins (HSPs) and branched-chain amino acid metabolism. In T2, ribosomal function (GO:0003735, *P adjust* < 0.01) and translation processes (GO:0006412, *P adjust* < 0.01) were explosively activated, driving peptide biosynthesis (GO:0043043, *P adjust* < 0.01) and nucleotide metabolism (GO:0032261, *P adjust* = 0.01). T3 marked a shift toward structural remodeling and metabolic reprogramming, with co-enrichment of cuticle formation (GO:0042302, *P adjust* < 0.01) and structural molecule activity (GO:0005198, *P adjust* < 0.01). In T4–T5, the pathways focused on reproductive maturation, including efficient coordination of eggshell formation (GO:0030703 *P adjust* < 0.01), ribosomal biogenesis (GO:0022626, *P adjust* < 0.01), cellular respiration (GO:0045333, *P adjust* < 0.01), and significant activation of germ cell maturation pathways (GO:0048477, *P adjust* < 0.01).

#### 3.2.3. KEGG Pathway Analysis

**Figure 6:**
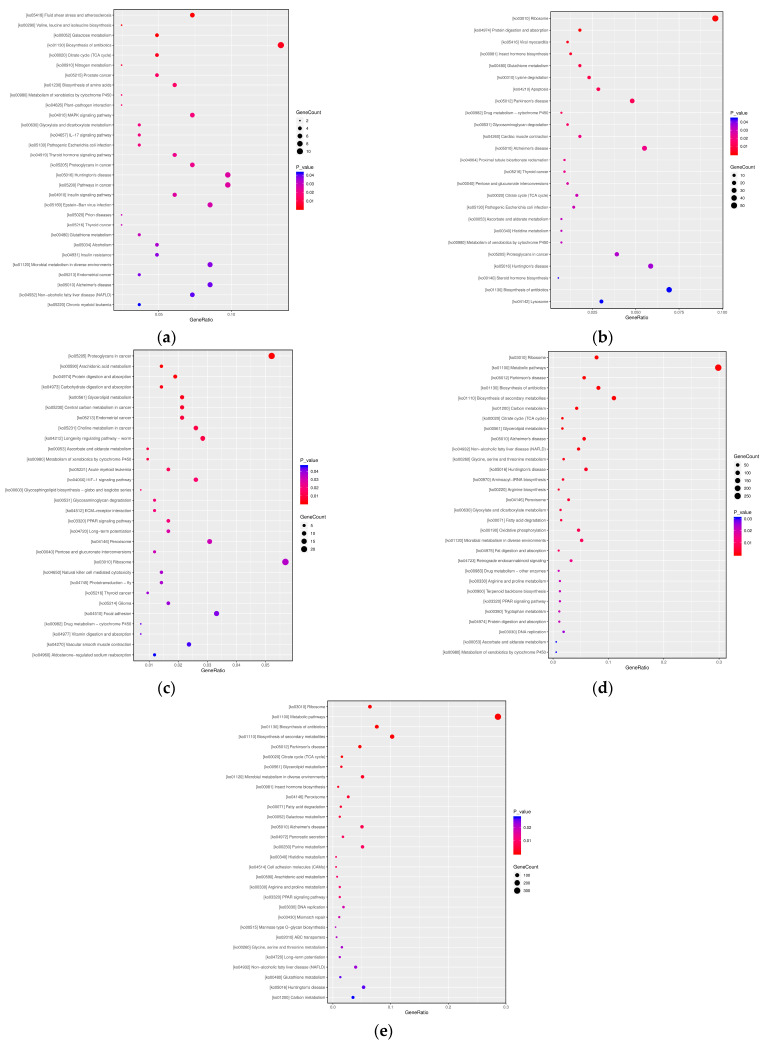
Transcriptome KEGG analysis of *A. pernyi* pupae at different developmental stages ((**a**): T1 vs. CK; (**b**): T2 vs. CK; (**c**): T3 vs. CK; (**d**): T4 vs. CK; (**e**): T5 vs. CK).

Integrated cross-stage analysis demonstrated stage-specific reprogramming of metabolic networks (Figure 6). In T1, energy metabolism (citrate cycle, ko00020, *P adjust* > 0.05) and detoxification pathways (glutathione metabolism, ko00052, *P adjust* > 0.05) were preliminarily activated but failed to reach significance after adjustment (*P adjust* > 0.05). T2 exhibited extreme enrichment of ribosomal pathways (ko03010, *P adjust* < 0.01), enhancing energy metabolism. T3 showed upregulated proteoglycan metabolism (ko05205, *P adjust* > 0.05), lipid metabolism (arachidonic acid metabolism, ko00590, *P adjust* > 0.05), and nutrient absorption pathways (*P adjust* = 0.20). In T4, ribosomal pathways (ko03010, *P adjust* < 0.01), metabolic pathways (ko01100, *P adjust* < 0.01), antibiotic biosynthesis (ko01130, *P adjust* < 0.01), and Parkinson’s disease-related pathways (ko05012, *P adjust* < 0.01) were significantly enriched. T5 similarly displayed marked differences in metabolic pathways and biological processes, including ribosomal activity, metabolic pathways, and antibiotic biosynthesis.

### 3.3. Integrated Analysis of Transcriptomics and Metabolomics

**Figure 7:**
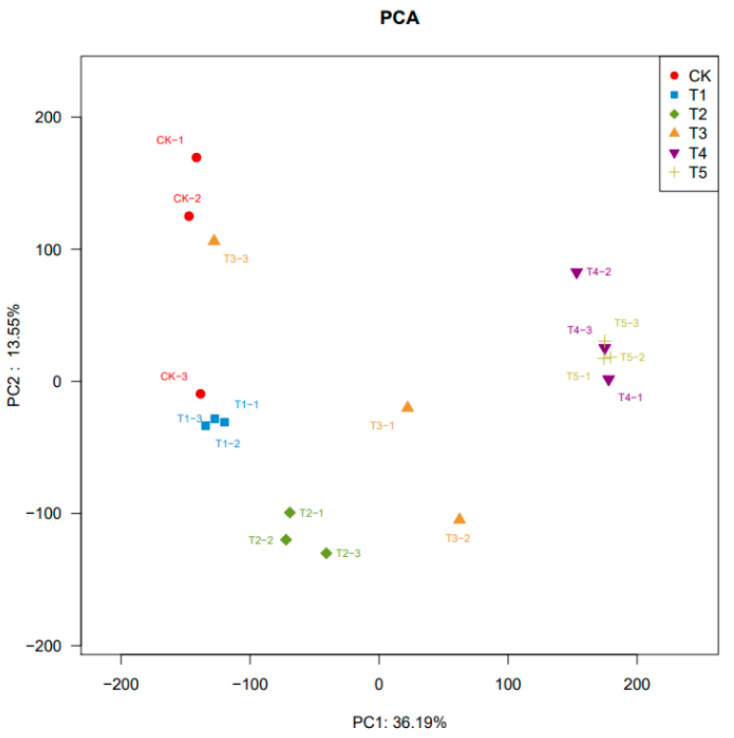
Changes in significantly differentially expressed genes.

RNA-seq analysis was performed on *A. pernyi* pupae at different developmental stages. In the PCA, PC1 showed a clear separation between the CK group and the T3 and T5 group samples, while in PC2, the samples from the T1, T3, and T5 groups were positioned adjacently (Figure 7). A total of 7230 differentially expressed genes (DEGs) were identified across the three comparison groups (T1 vs. CK, T3 vs. CK, and T5 vs. CK). The expression levels of 366, 1705, and 5159 significantly differentially expressed genes (DEGs) in each comparison group (T1 vs. CK, T3 vs. CK, and T5 vs. CK), respectively.

**Figure 8:**
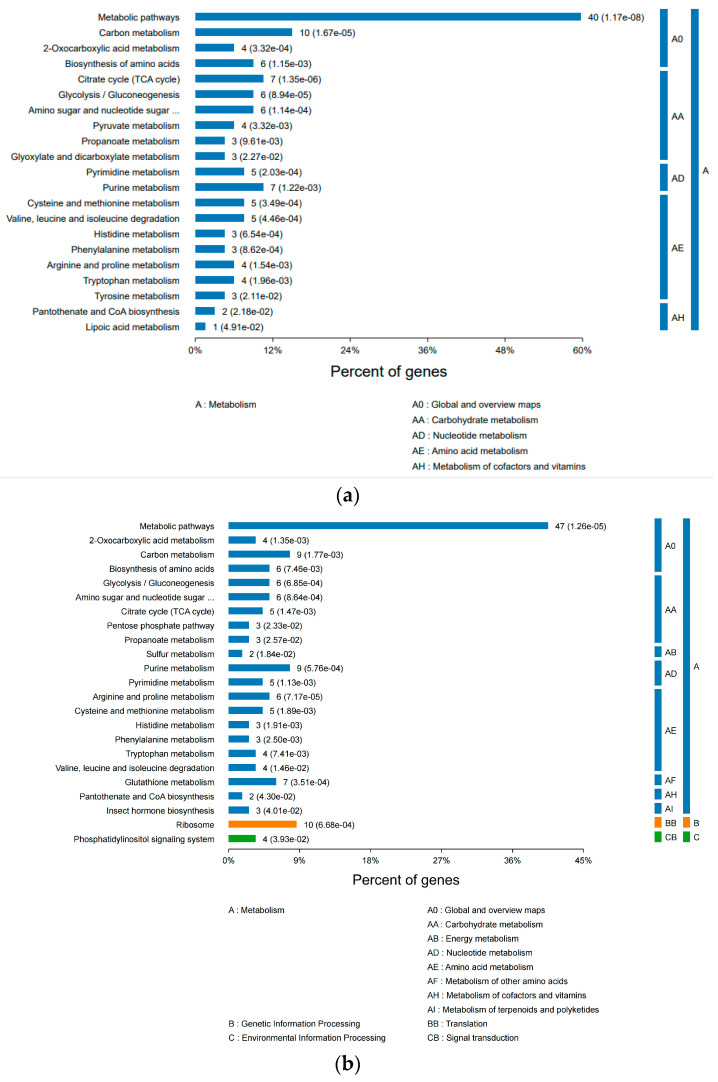

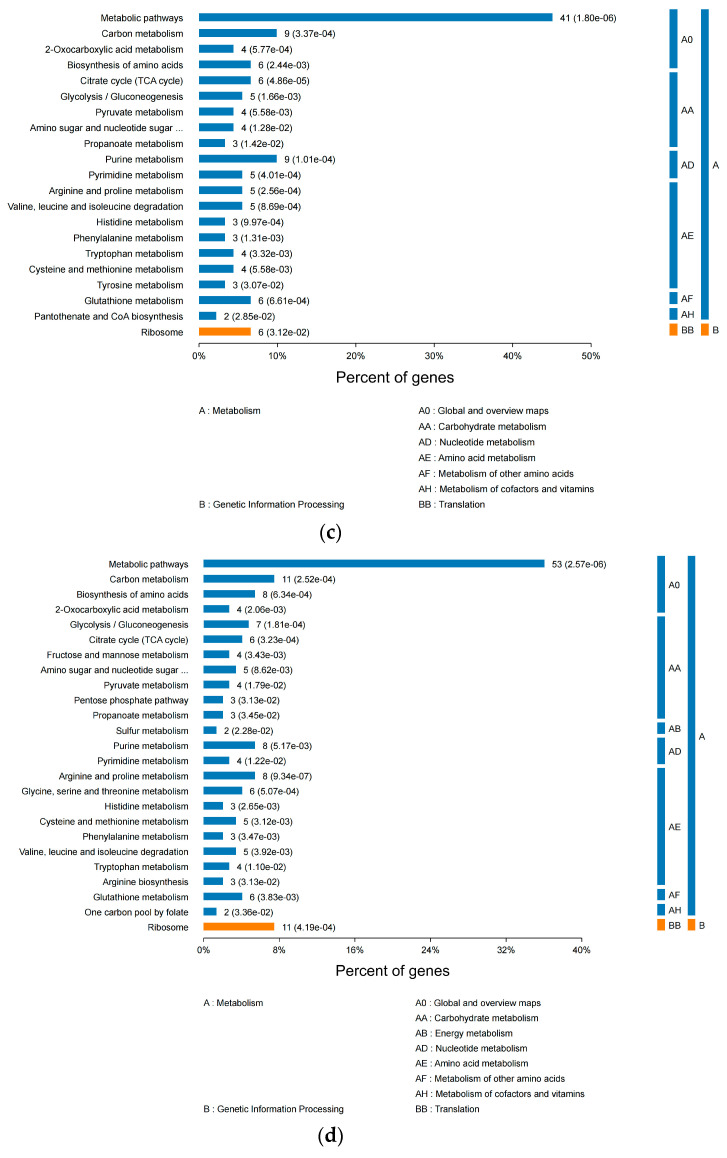

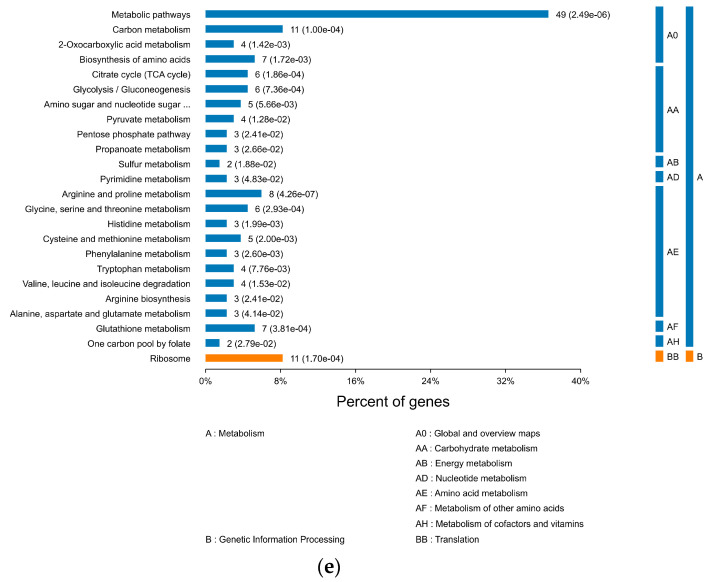
Integrated protein–metabolite KEGG pathway enrichment analysis ((**a**): T1 vs. CK; (**b**): T2 vs. CK; (**c**): T3 vs. CK; (**d**): T4 vs. CK; (**e**): T5 vs. CK).

The developmental stages T1–T5 of *A. pernyi* pupae exhibit distinct, dynamic changes in metabolic pathways (Figure 8): In the early and mid-early stages (T1–T2), carbon metabolism, TCA cycle, and amino acid biosynthesis dominate (*p* < 0.01), providing energy and building blocks for tissue reconstruction. During the mid-stage (T3), TCA cycle activity peaks (*p* = 4.86 × 10^−5^) while ribosomal pathways become activated (*p* = 3.12 × 10^−2^), marking the initiation of adult organ development. In mid-late and later stages (T4–T5), the metabolic focus shifts toward protein synthesis (ribosome *p* = 1.70 × 10^−4^) and stress response (glutathione metabolism *p* = 3.81 × 10^−4^), with significant enhancement of gluconeogenesis pathways (*p* = 7.36 × 10^−4^), indicating preparation for eclosion. Notably, the stage-specific activation of the pentose phosphate pathway (T2) and one-carbon metabolism (T5) reflects the specialized roles of NADPH demand and epigenetic regulation during developmental transitions. These metabolic reprogramming events systematically support the morphological construction and physiological functional transformation from pupa to adult.

## 4. Discussion

The integration of multi-omics approaches has provided a comprehensive understanding of the dynamic metabolic and transcriptional changes during the development of *A. pernyi* pupae. Our findings reveal that nutrient composition and bioactive compound accumulation are tightly regulated across the developmental stages, driven by coordinated shifts in key metabolic pathways and gene expression patterns. These insights align with recent advancements in insect developmental biology and multi-omics integration, offering novel perspectives on the molecular mechanisms underlying nutrient metabolism in lepidopteran species.

### 4.1. Metabolic Reprogramming and Nutrient Dynamics

The metabolomic profiling identified 1246 metabolites, with significant shifts in amino acids, pyrimidines, and monosaccharides across five developmental stages. The decline in free amino acids (e.g., proline, lysine) and the concomitant rise in pyrimidine derivatives (e.g., uridine, β-alanine) and monosaccharides (e.g., glucose, fructose) suggest a metabolic transition from protein synthesis to energy production and nucleic acid metabolism during pupal maturation. These observations are consistent with studies in *Bombyx mori*, where nutrient reserves are repurposed to fuel metamorphosis and tissue remodeling [13]. Notably, the upregulation of tyrosine and methionine in later stages aligns with their roles as precursors for cuticle sclerotization and antioxidant synthesis, critical for surviving environmental stressors [18]. The accumulation of 18β-glycyrrhetinic acid and ursolic acid in the mid to late stages highlights the potential of *A. pernyi* pupae as a source of triterpenoids, which exhibit anti-inflammatory and immunomodulatory properties [23]. The extraordinary accumulation of 18β-glycyrrhetinic acid (1677-fold) and ursolic acid (13,126-fold) in the mid to late stages (T4–T5) likely reflects a dual biological imperative. Firstly, these triterpenoids serve as potent anti-inflammatory and antioxidant agents, crucial for mitigating the oxidative stress associated with extensive tissue remodeling during late pupal development and preparation for eclosion. Secondly, their accumulation may represent a strategic reservoir of bioactive precursors for adult cuticle sclerotization and innate immune defense upon emergence, mirroring patterns observed in other holometabolous insects where specialized metabolites peak prior to critical developmental transitions.

The T1-stage metabolic profile aligns with the energy demands of early metamorphosis, where nitrogen-rich amino acids (e.g., glutamate) and pyrimidines likely support tissue remodeling [24]. The persistent activation of the TCA cycle across the T1–T5 stages suggests its dual role in energy provision and biosynthetic precursor supply [25]. The T2-stage dominance of ABC transporters and ribosomes mirrors developmental transitions in holometabolous insects, where transmembrane transport and protein synthesis are critical for organogenesis [26,27]. The T3-stage glutathione surge (1444.5-fold) and MAPK activation imply a systemic oxidative stress response, potentially triggered by mitochondrial ROS leakage during rapid growth [28]. In T4, the interaction between LOC genes and TCA intermediates (e.g., 2-ketoglutarate) may reflect a regulatory nexus linking mitochondrial metabolism to epigenetic modifications [29]. The extreme glutathione accumulation in T5 (1976.56-fold) underscores its role not only in redox buffering but also as a signaling molecule modulating apoptosis and immune responses [30].

### 4.2. Transcriptional Regulation of Developmental Transitions

Transcriptomic analysis revealed 7230 differentially expressed genes (DEGs), with stage-specific enrichment in pathways such as ABC transporters, amino acid biosynthesis, and pyrimidine metabolism. The progressive increase in DEGs from T1 to T5 underscores the escalating complexity of transcriptional regulation as development advances. For instance, the upregulation of ABC transporters in later stages correlates with enhanced nutrient transport and detoxification, a mechanism also observed in *Drosophila melanogaster* to mitigate oxidative stress during metamorphosis [31]. The enrichment of aminoacyl-tRNA biosynthesis in mid to late stages further emphasizes the demand for translational machinery to support rapid tissue differentiation. These findings echo recent work on *Manduca sexta*, where dynamic tRNA pools regulate metamorphic progression [32]. Among the 5159 DEGs identified in T5, genes exhibiting the most pronounced upregulation included those encoding heat shock proteins (e.g., Hsp70, Hsp90), glutathione S-transferases (GSTs), and ribosomal proteins. The significant induction of Hsp70 and Hsp90 aligns with their well-documented roles in lepidopteran metamorphosis as molecular chaperones facilitating protein folding and stability under developmental stress [33]. Similarly, the upregulation of GSTs corroborates findings in *Bombyx mori* and *Manduca sexta*, where these enzymes are essential for detoxification and redox homeostasis during pupal–adult transformation [14,34]. The coordinated surge in ribosomal protein genes further supports the heightened demand for de novo protein synthesis underlying adult organogenesis, a conserved feature across insect metamorphosis [35].

Early-stage stress adaptation (GO:0010998) and cellular homeostasis (GO:0045214) align with conserved strategies in insect metamorphosis [35]. The global activation of ribosome pathways (GO:0003735) during T2 supports organogenesis through material synthesis, paralleling mechanisms in Drosophila pupae [36]. Late-stage synergy between germ cell maturation (GO:0048477) and ribosome biogenesis (GO:0022626) underscores the spatiotemporal regulation of protein synthesis machinery in developmental transition [37].

KEGG analysis of *A. pernyi* pupae across the T1–T5 stages highlights the dynamic regulation of metabolism and disease-related pathways during development. Stage-specific enrichment of branched-chain amino acid synthesis (T1), ribosomal activity (T2 and T4), and fatty acid metabolism (T3) reflects distinct metabolic priorities, including energy supply, protein synthesis, and lipid storage. The enrichment of human disease-related pathways (e.g., Parkinson’s and Alzheimer’s diseases) likely mirrors conserved biological processes in pupal development, such as oxidative stress response and proteostasis maintenance. Although the significance of certain pathways requires further validation, this study provides novel insights into the molecular mechanisms of *A. pernyi* metamorphosis and the evolutionary conservation of insect–human regulatory networks. It also lays a theoretical foundation for biotechnological applications, such as antimicrobial peptide development.

### 4.3. Multi-Omics Convergence and Biological Implications

Integrated analysis revealed compelling gene–metabolite concordance. For instance, the significant upregulation of ABC transporter genes (e.g., GO:0043190) in the T4–T5 stages directly paralleled the accumulation of their putative substrates, including glucose, mannose, and N-acetylglucosamine. Similarly, the coordinated downregulation of genes encoding key enzymes in lysine and proline biosynthesis (e.g., *dapA*, *p5cR*) mirrored the decline in these amino acid metabolites across development. Notably, the persistent upregulation of tyrosine across stages was underpinned by the sustained expression of tyrosine aminotransferase (GO:0070547), linking transcriptional control to metabolic flux in cuticle sclerotization pathways.

The integration of metabolomic and transcriptomic data highlights the centrality of ABC transporters in shuttling nutrients (e.g., sugars, amino acids) and xenobiotics, a finding corroborated by recent studies in *Tribolium castaneum* [38]. The coordinated downregulation of amino acid biosynthesis genes and corresponding metabolites suggests a resource allocation strategy favoring energy storage over growth, a hallmark of insect diapause termination [19]. Furthermore, the accumulation of triterpenoids like ursolic acid implicates these compounds in enhancing pupal survivability, possibly through antimicrobial or antioxidative mechanisms, as demonstrated in Galleria mellonella [39].

### 4.4. Limitations and Future Directions

While this study provides a robust framework for understanding *A. pernyi* pupae development, limitations include the absence of functional validation (e.g., gene knockout) and temporal resolution within stages. Future work should employ single-cell RNA sequencing to resolve tissue-specific metabolic heterogeneity, CRISPR-Cas9 editing to verify the roles of key DEGs, and RNA interference (RNAi) targeting key candidate genes identified here (e.g., specific ABC transporters like *ABCG-X* or aminoacyl-tRNA synthetases) in *A. pernyi* pupae. Knockdown at defined developmental stages (e.g., T3–T4) followed by metabolomic profiling and assessment of phenotypic outcomes (e.g., eclosion success, stress susceptibility, metabolite levels of β-alanine, ursolic acid) will provide direct functional validation of their roles in nutrient metabolism and developmental progression, moving beyond correlative insights. Furthermore, enzyme activity assays should quantify key catalytic nodes in enriched pathways, such as tyrosine aminotransferase (TAT) for cuticle sclerotization, dihydroorotate dehydrogenase (DHODH) for pyrimidine synthesis, and ABC transporter ATPase for nutrient shuttling efficiency. These assays would directly correlate transcriptional/metabolomic shifts (e.g., sustained tyrosine accumulation in Figure 4; 20-fold 4-methylpyrimidine upregulation in T5) with biochemical functionality, establishing causal links to observed developmental phenotypes like pupal tanning and nutrient reservoir formation. Additionally, expanding the metabolomic database to include lipidomic and hormone profiles could elucidate crosstalk between nutrient metabolism and endocrine signaling.

## 5. Conclusions

During the development of *A. pernyi* pupae, the profiles of bioactive substances exhibit significant stage-specific variations, reflecting dynamic metabolic and regulatory adaptations. In the early stage (T1), compared with the control group (CK), there are five upregulated and six downregulated amino acid metabolites, three upregulated and six downregulated pyrimidine metabolites, and six upregulated and six downregulated metabolites in the ABC transporter pathway. By the middle stage (T3), metabolic shifts become more pronounced, with 2 amino acid metabolites upregulated and 10 downregulated, 6 pyrimidine metabolites upregulated and 5 downregulated, and 6 metabolites in the ABC transporter pathway upregulated while 16 are downregulated. Concurrently, the expression of Hsp83 and Hsp90b1 in the NOD-like receptor signaling pathway is upregulated, indicating an active stress response. In the late stage (T5), 7 amino acid metabolites are upregulated and 11 downregulated, 8 pyrimidine metabolites are upregulated and 2 downregulated, and 14 metabolites in the ABC transporter pathway are upregulated while 12 are downregulated. Additionally, proteins associated with sugar metabolism and immune response are significantly upregulated, while the expression of Hsp83, Hsp90b1, and antioxidant enzymes declines. Notably, the accumulation of 18β-glycyrrhetinic acid and ursolic acid becomes significant during this stage.

The developmental progression of *A. pernyi* pupae is characterized by tightly regulated metabolic and stress responses with distinct patterns at each stage. These changes ensure efficient resource allocation, stress adaptation, and immune preparedness, ultimately supporting successful development and survival. The accumulation of specific bioactive compounds, such as 18β-glycyrrhetinic acid and ursolic acid, highlights the intricate balance between metabolic and defense mechanisms during pupal development. Future studies should focus on elucidating the functional roles of these compounds and exploring their potential applications in biotechnology and medicine.

This multi-omics study delineates the molecular landscape of *A. pernyi* pupae development, revealing stage-specific metabolic and transcriptional signatures that underpin nutrient dynamics. The findings not only advance our knowledge of lepidopteran physiology but also provide a foundation for exploiting pupae as sustainable sources of bioactive compounds for nutraceutical and pharmaceutical applications.

## Figures and Tables

**Table 1:** Classification and proportion of metabolites in *A. pernyi* pupae.

Classification	Number	Proportion
Organic acids and derivatives	135	24.46%
Organic heterocyclic compounds	102	18.48%
Lipids and lipid-like molecules	73	13.22%
Organic oxygen-containing compounds	64	11.59%
Phenylpropanoids and polyketides	63	11.41%
Benzene-type compounds	60	10.87%
Nucleosides, nucleotides, and derivatives	32	5.80%
Alkaloids and derivatives	7	1.27%
Organic nitrogen compounds	7	1.27%
Lignans, neolignans, and related compounds	5	0.91%
Homogeneous non-metal compounds	2	0.36%
Derivatives of hydrocarbons	1	0.18%
Organic sulfur compounds	1	0.18%

**Table 2:** Comparison of the number of differential metabolites in *A. pernyi* pupae at different developmental stages.

Group	Total	Upregulated	Downregulated
T1 vs. CK	424	146	278
T2 vs. CK	509	243	266
T3 vs. CK	564	276	288
T4 vs. CK	647	356	291
T5 vs. CK	710	411	299

## Data Availability

The data presented in this study are available on request from the corresponding author due to privacy and legal reasons.

## Supplementary Materials

The following supporting information can be downloaded at: https://www.mdpi.com/article/10.3390/insects16070745/s1, The original Figure 3 are available in the Supplementary Materials.
