# Supplementary figures and images for: Integrated Multi-Omics Analysis of the Developmental Stages of Antheraea pernyi Pupae: Dynamic Changes in Metabolite Profiles and Gene Expression
Source: Insects. 2025 Jul 21;16(7):745. doi: 10.3390/insects16070745 (PMC12295449; doi:10.3390/insects16070745)

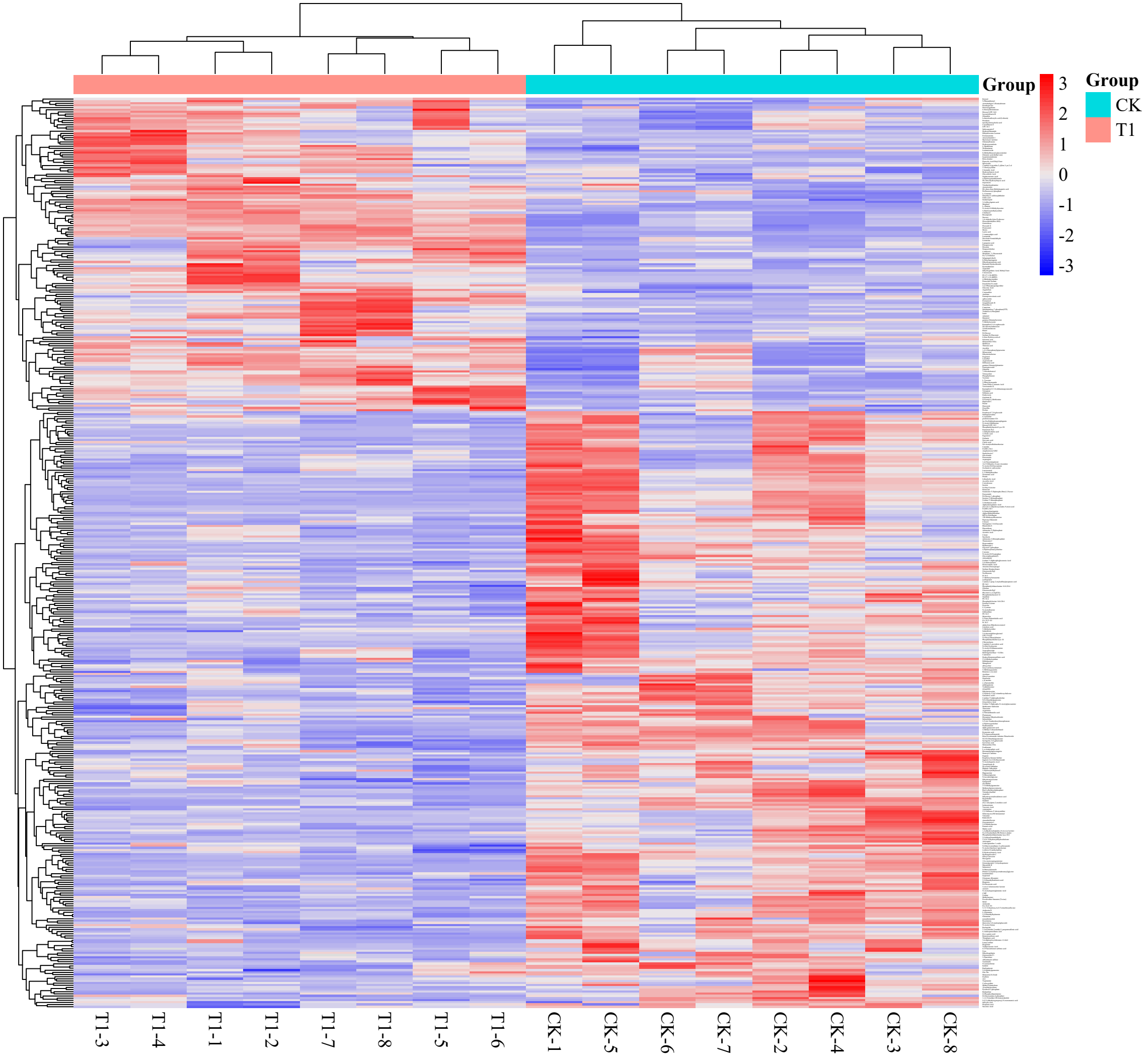

Supplement: Supplementary file 1 [file insects-16-00745-s001.zip › Figure 3/Figure3 T1 vs CK.pdf]

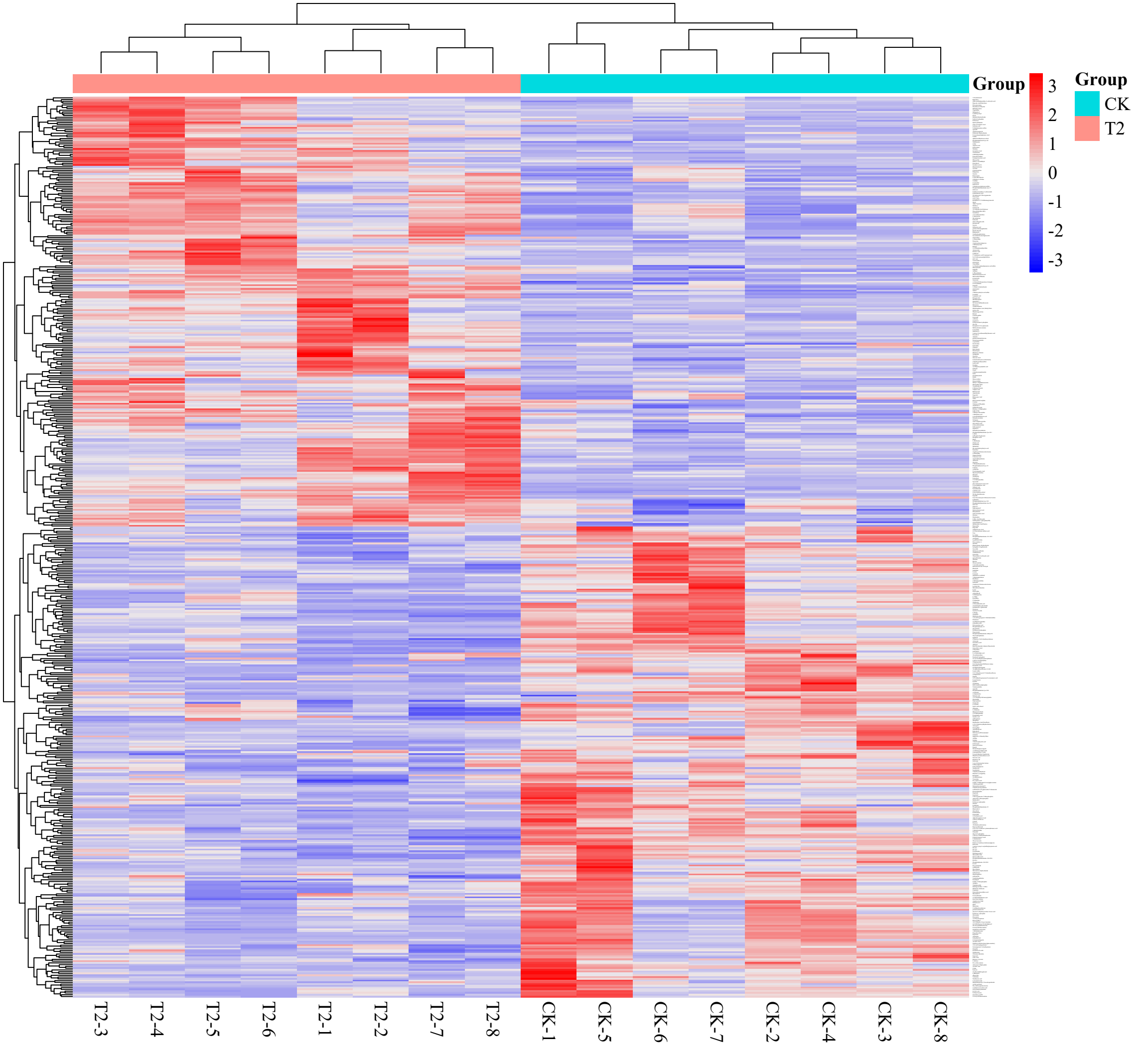

Supplement: Supplementary file 1 [file insects-16-00745-s001.zip › Figure 3/Figure3 T2 vs CK.pdf]

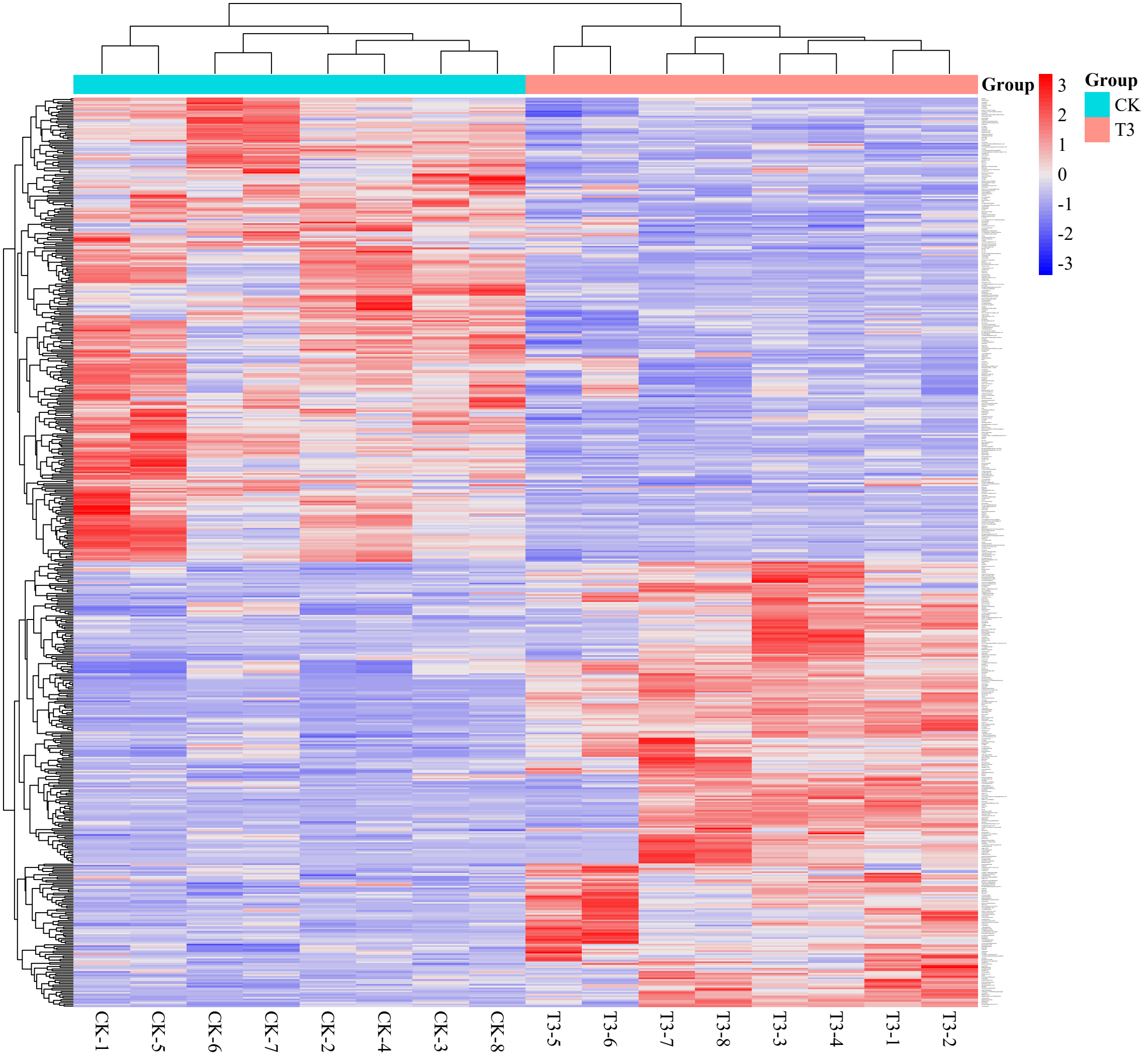

Supplement: Supplementary file 1 [file insects-16-00745-s001.zip › Figure 3/Figure3 T3 vs CK.pdf]

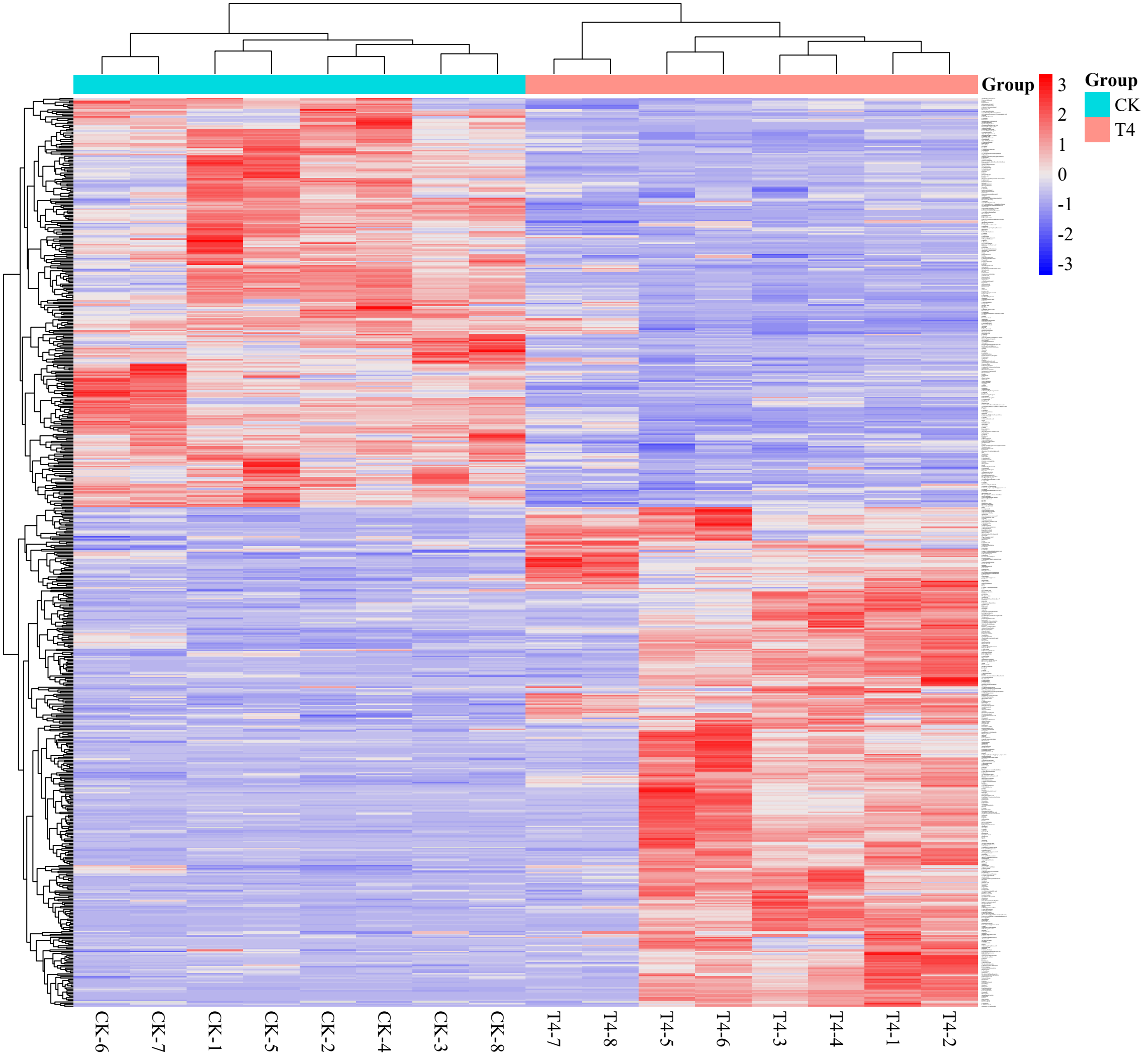

Supplement: Supplementary file 1 [file insects-16-00745-s001.zip › Figure 3/Figure3 T4 vs CK.pdf]

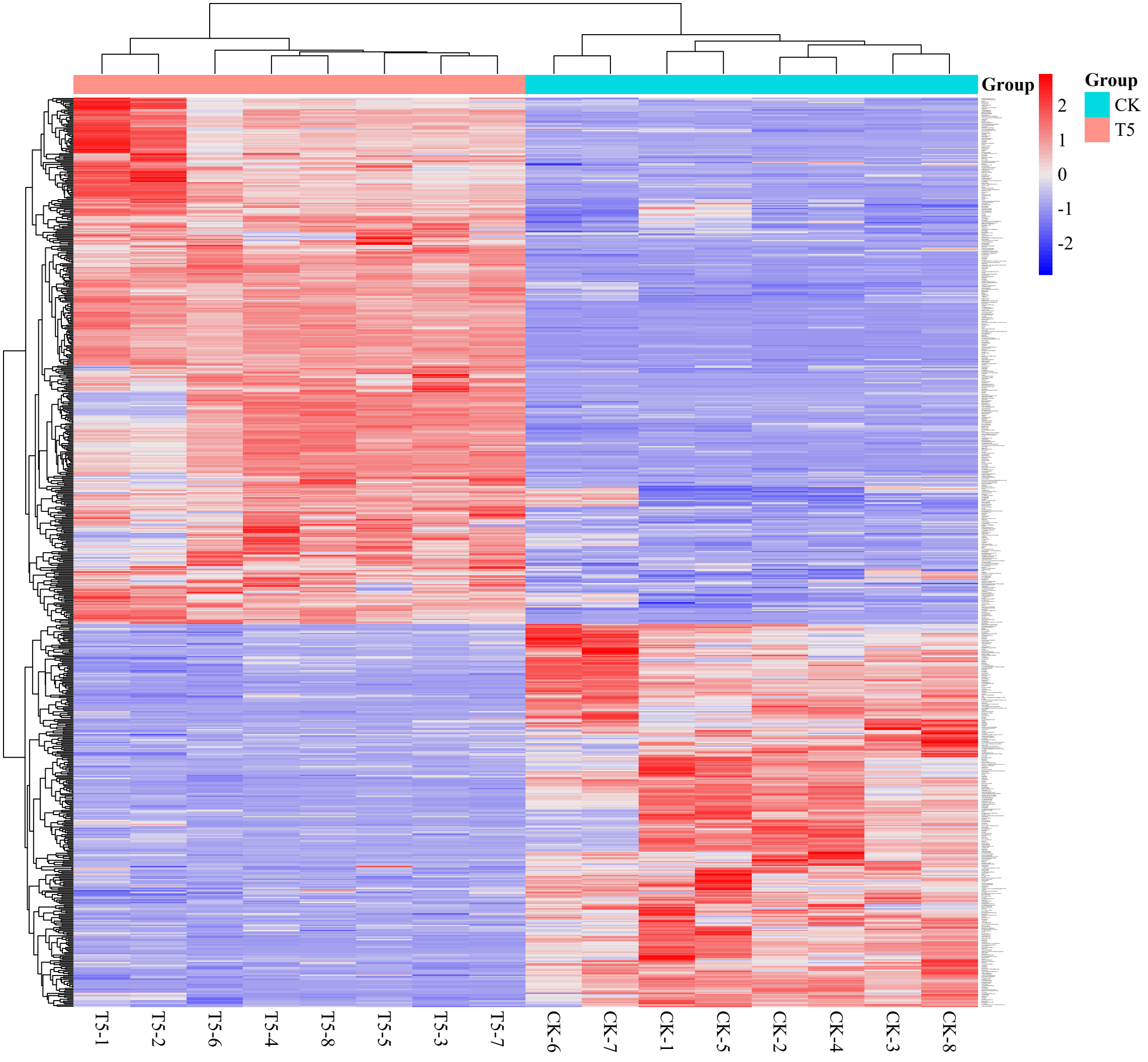

Supplement: Supplementary file 1 [file insects-16-00745-s001.zip › Figure 3/Figure3 T5 vs CK.pdf]
